# Protocol for identifying components of subcellular compartments by antibody-based *in situ* biotinylation

**DOI:** 10.1016/j.xpro.2025.103803

**Published:** 2025-04-30

**Authors:** Hidefumi Suzuki, Kexin Li, Sayaka Ito, Keiichiro Asano, Keisuke Noguchi, Ryota Abe, Hidehisa Takahashi

**Affiliations:** 1 Department of Molecular Biology, Yokohama City University Graduate School of Medical Science, 3-9 Fukuura, Kanazawa-ku, Yokohama, Kanagawa 236-0004, Japan

**Keywords:** Cell biology, Molecular biology, Proteomics

## Abstract

Recent studies revealed that membrane-less subcellular organelles play important roles in cellular functions. Here, we present a protocol for identifying subcellular compartment components by antibody-based *in situ* biotinylation. We describe steps for *in situ* biotinylation labeling using a horseradish peroxidase (HRP)-conjugated antibody, purification of the biotinylated components, and sample preparation for high-throughput analysis. This protocol has potential for application in the comprehensive analysis of dynamic subcellular organelles.

For complete details on the use and execution of this protocol, please refer to Noguchi et al.^1^

## Highlights

• Steps for *in situ* biotinylation to identify the components of nuclear bodies
• Instructions on identifying DNAs, RNAs, and proteins in nuclear bodies
• Procedures for preparing biotinylated proteins for MS or DNAs/RNAs for NGS

## Abstract (editor highlights)

Publisher’s note: Undertaking any experimental protocol requires adherence to local institutional guidelines for laboratory safety and ethics.

## Graphical abstract

**Figure:**
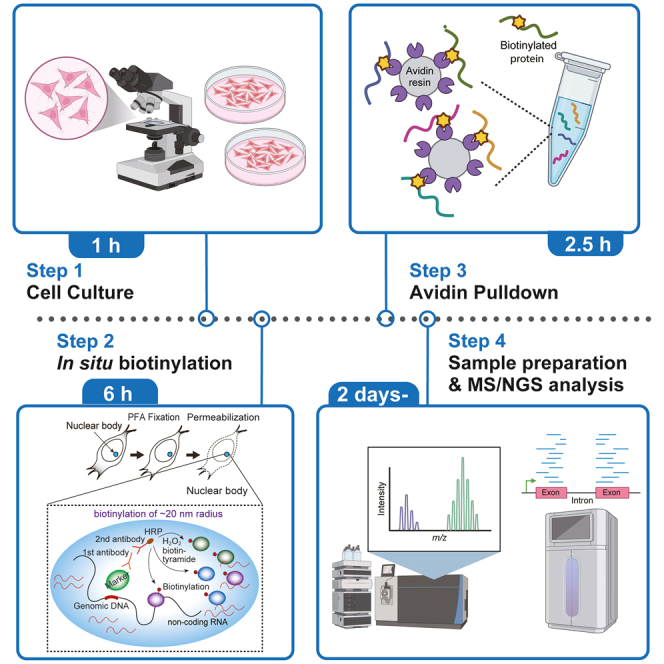

## Before you begin

**Figure 1:**
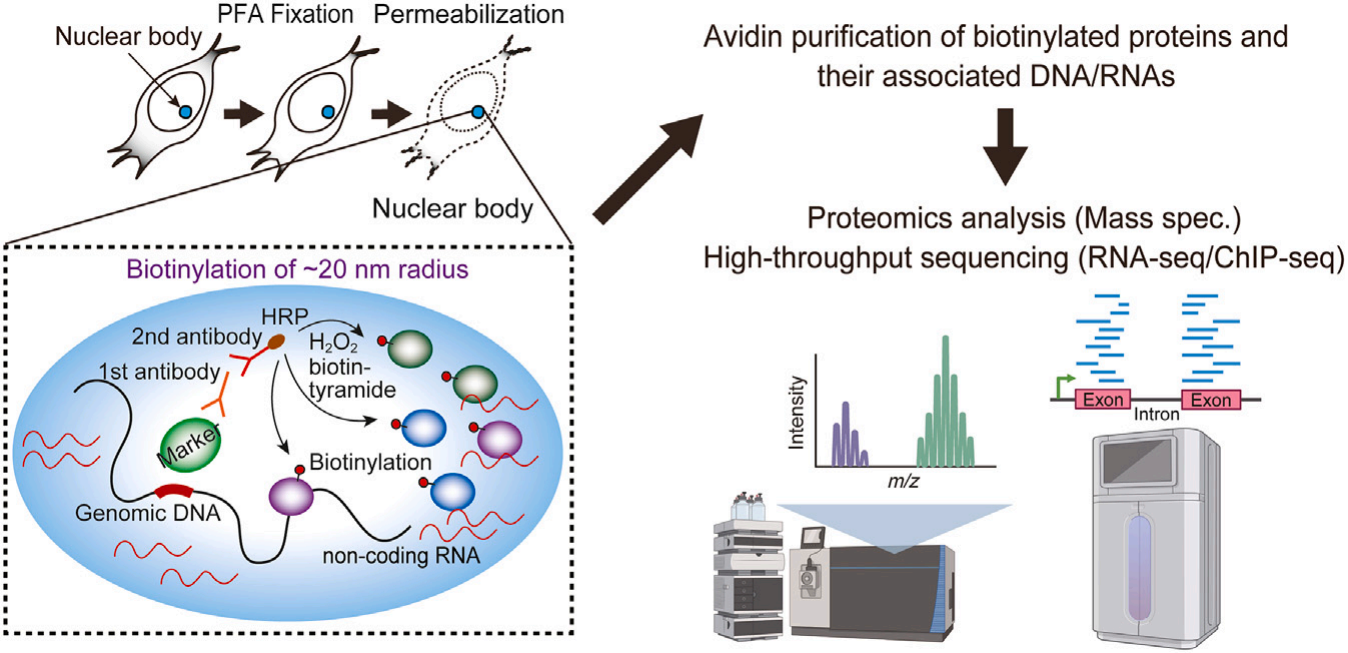
Antibody-based *in situ* biotinylation method for detection of nuclear body components In this antibody-based *in situ* biotinylation strategy, HRP is exogenously delivered to target proteins in fixed and permeabilized cells using an antibody recognizing the target protein. For HRP-based *in situ* biotinylation, incubate the cells with biotin phenol and H_2_O_2_. The biotinylated components can be purified by avidin pulldown, and purified proteins or bound genomic DNA and RNA can be comprehensively analyzed by high-throughput analysis.

Membrane-less subcellular compartments, including cytoplasmic bodies, nuclear bodies, paraspeckles, and other liquid droplets formed by liquid-liquid phase separation (LLPS), play important roles in many cellular functions.^2^^,^^3^ Comprehensive identification of components of such membrane-less compartments helps in understanding their functions and physiological roles. Several proximity-based labeling methods, including biotin ligase-dependent labeling (e.g., BioID, TurboID), engineered ascorbate peroxidase (APEX)-based labeling, and horseradish peroxidase (HRP)-based labeling, have been developed to identify the protein components of these compartments.^4^^,^^5^^,^^6^^,^^7^^,^^8^^,^^9^^,^^10^^,^^11^^,^^12^ In the HRP-based strategy, cultured cells are fixed, permeabilized, and subjected to *in situ* binding of HRP-conjugated secondary antibodies. HRP-based *in situ* biotinylation is performed by incubating the cells with biotin phenol and H_2_O_2_. The resulting biotinylated components are pulled down using avidin beads, and purified proteins or bound genomic DNA and RNA are analyzed by mass spectrometry or next-generation sequencing, respectively (Figure 1). This antibody-based proximity labeling has several advantages over the other methods. First, PFA fixation allows us to obtain snapshots with more temporal resolution than the other proximity labeling methods. Second, it is not necessary to exogenously express biotin ligase- or peroxidase-fusion proteins. Third, this method is applicable to proximity identification of post translational modifications, which is not possible with the other methods. The success of this technique depends highly on the performance of antibodies, so it is very important to consider the performance and specificity of the antibody before you begin. The protocol below describes the steps to prepare samples for protein analysis by mass spectrometry and DNA/RNA analysis by NGS with HeLa cells, HCT116 cells, and HEK293T cells. Under optimized experimental conditions, this technique can also be applied to other adherent cells.

### Preparation of stock solutions

#### 16% paraformaldehyde (50 mL)

Dissolve 8 g of PFA in 30 mL of MilliQ water. Add 600 μL of 1 N NaOH, and heat at 65°C for 20 min. Add 5 mL of 10× concentration PBS, and adjust the pH to 7.0–8.0. Add MilliQ water up to 50 mL. Then, filter with a 0.45 μm PVDF membrane filter.

**CRITICAL:** PFA is harmful. Do not handle without gloves.

#### 50 mM biotin phenol (1 mL)

Dissolve 18 mg of Biotin phenol in 1 mL of DMSO. Biotin phenol, MW: 363.47 g/mol.

#### 1X TE (50 mL)

Prepare a solution of 10 mM Tris-HCl (pH 7.5) and 1 mM EDTA in 50 mL of MilliQ water. Add 0.5 mL of 1 M Tris-HCl (pH 7.5) and 0.1 mL of 0.5 M EDTA in 49.4 mL of MilliQ water.

#### 10% sodium dodecyl sulfate (50 mL)

Dissolve 5 g of SDS in 50 mL of MilliQ water. SDS, MW: 288.38 g/mol.

#### 10% sodium deoxycholate (50 mL)

Dissolve 5 g of Doc in 50 mL of MilliQ water. Doc, MW: 414.55 g/mol.

#### 10% Triton X-100 (50 mL)

Combine 5 mL of Triton X-100 and 45 mL of MilliQ water.

#### 10% bovine serum albumin (50 mL)

Dissolve 5 g of BSA in 50 mL of PBS.

#### 5 M NaCl (100 mL)

Dissolve 29.22 g of NaCl in 100 mL of MilliQ water. NaCl, MW: 58.44 g/mol.

## Key resources table

**Table:** 

REAGENT or RESOURCE	SOURCE	IDENTIFIER
**Antibodies**		

Anti-rabbit IgG, HRP-conjugated (use at 1:4,000 dilution)	Cell Signaling Technology	Cat#7074
Anti-mouse IgG, HRP-conjugated (use at 1:4,000 dilution)	Cell Signaling Technology	Cat#7076
Mouse monoclonal anti-Coilin (use at 1:2,000 dilution)	Abcam	Cat#11822
Mouse monoclonal anti-phospho-Histone H2A.X (Ser139) clone JBW301 (use at 1:500 dilution)	Sigma-Aldrich	Cat#05-636-1
Mouse monoclonal anti-β actin (use at 1:5,000 dilution)	Santa Cruz Biotechnology	Cat#sc-47778
Goat anti-mouse IgG (H + L) highly cross-adsorbed secondary antibody, Alexa Fluor 488 (use at 1:2,000 dilution)	Thermo Fisher Scientific	A-11029

**Chemicals, peptides, and recombinant proteins**		

Biotin phenol	Iris Biotech	LS-3500
Sodium dodecyl sulfate (SDS)	Sigma-Aldrich	L3771
Sodium deoxycholate (Doc)	FUJIFILM Wako	194-08311
Sodium chloride (NaCl)	Nacalai Tesque	31319-45
Ammonium hydrogencarbonate (NH_4_HCO_3_)	Nacalai Tesque	08887-54
Iodoacetamide (IAA)	FUJIFILM Wako	093-02152
Trypsin Gold, mass spectrometry grade	Promega	V5280
Dithiothreitol (DTT)	Nacalai Tesque	14112-94
Acetonitrile hypergrade for LC-MS (I)	Merck	1.00029.1000
Trifluoroacetic acid for protein sequence analysis (TFA)	Merck	1.08178.0050
Distilled water	Nacalai Tesque	14029-91
Hydrogen peroxide (H_2_O_2_) (30%)	FUJIFILM Wako	081-04215
Triton X-100	Nacalai Tesque	12969-25
Paraformaldehyde	Merck Millipore	1.04005.1000
TRIzol LS reagent	Thermo Fisher Scientific	10296028
GlycoBlue coprecipitant	Thermo Fisher Scientific	AM9516
SoftLink soft release avidin resin	Promega	9PIV201
Dulbecco’s phosphate-buffered saline (PBS)	Nacalai Tesque	14249-24
Proteinase K	Nacalai Tesque	29442
Protease inhibitor cocktail (EDTA free)	Nacalai Tesque	03969
Ribonuclease A	Nacalai Tesque	30141-14
Streptavidin HRP	Cytiva	RPN1231-2ML
Streptavidin, Alexa Fluor 647 conjugate	Thermo Fisher Scientific	S32357’
4′,6-diamidino-2-phenylindole dihydrochloride (DAPI)	Nacalai Tesque	1034-56
Cellmatrix type I-C, collagen	Nitta Gelatin	N/A
ProLong glass antifade mountant	Thermo Fisher Scientific	P36984
SUPERase·In RNase inhibitor	Thermo Fisher Scientific	AM2694

**Critical commercial assays**		

QIAquick PCR purification kit	QIAGEN	28106
Bioruptor sonicator	Diagenode	N/A
NEBNext Ultra II DNA library prep kit for Illumina	Illumina	E7645
NEBNext Ultra II directional RNA library prep kit for Illumina	Illumina	E7760

**Experimental models: Cell lines**		

Human: HeLa	ATCC	CCL-2
Human: HCT116	ATCC	CCL-247

**Other**		

Micro cover glasses, round 15 mm, 0.13–0.17 mm	MATSUNAMI	N/A
Tissue culture dish easy-grip 60 × 15 style	Corning	353004
Tissue culture dish easy-grip 100 × 20 style	Corning	353003
Tissue culture dish easy-grip 35 × 10 style	Corning	353001
CELLSTAR multiwell plate, 12 well	Greiner Bio-One	665180
Dulbecco’s modified Eagle’s medium (DMEM)	Nacalai Tesque	08458
Bovine serum albumin	Nacalai Tesque	01863-48
Horse serum, New Zealand origin	Thermo Fisher Scientific	16050130
Centrifuge adapter	GL Sciences	5010-21514

## Materials and equipment

**Timing: variable**

***Note:*** Prepare stock solutions before starting experiments. All reagents should be filtered using 0.22 μm PVDF filters.

**Table:** Blocking buffer

Reagent	Final concentration	Amount
10% Bovine serum albumin	5%	5 mL
Horse serum (100%)	2%	0.2 mL
H_2_O	N/A	4.8 mL
**Total**	**N/A**	**10 mL**

Store at 4°C for up to 1 day

**Table:** Biotinylation buffer

Reagent	Final concentration	Amount
50 mM Biotin phenol	500 μM	100 μL
30% H_2_O_2_	0.0015%	0.5 μL
1X PBS	N/A	up to 10 mL
**Total**	**N/A**	**10 mL**

Prepare just before use. Scale up according to the size and number of samples.

***Note:*** Optimize the final concentration of Biotin phenol and H_2_O_2_ for each experiment.

**Table:** 1% SDS RIPA buffer

Reagent	Final concentration	Amount
1 M Tris-HCl (pH 8.0)	50 mM	2.5 mL
5 M NaCl	150 mM	1.5 mL
10% SDS	1%	5 mL
10% Triton X-100	1%	5 mL
10% Doc	0.5%	2.5 mL
H_2_O	N/A	33.5 mL
**Total**	**N/A**	**50 mL**

Store at 4°C for up to 3 months

**Table:** 2× Dilution buffer

Reagent	Final concentration	Amount
1 M Tris-HCl (pH 8.0)	50 mM	2.5 mL
5 M NaCl	150 mM	1.5 mL
10% Triton X-100	1%	5 mL
10% Doc	0.5%	2.5 mL
H_2_O	N/A	38.5 mL
**Total**	**N/A**	**50 mL**

Store at 4°C for up to 3 months

**Table:** 0.5% SDS RIPA buffer

Reagent	Final concentration	Amount
1 M Tris-HCl (pH 8.0)	50 mM	2.5 mL
5 M NaCl	150 mM	1.5 mL
10% SDS	0.5%	2.5 mL
10% Triton X-100	1%	5 mL
10% Doc	0.5%	2.5 mL
H_2_O	N/A	36 mL
**Total**	**N/A**	**50 mL**

Store at 4°C for up to 3 months

**Table:** 0.5 M NaCl RIPA buffer

Reagent	Final concentration	Amount
1 M Tris-HCl (pH 8.0)	50 mM	2.5 mL
5 M NaCl	0.5 M	5 mL
10% SDS	0.1%	0.5 mL
10% Triton X-100	1%	5 mL
10% Doc	0.5%	2.5 mL
H_2_O	N/A	34.5 mL
**Total**	**N/A**	**50 mL**

Store at 4°C for up to 3 months

**Table:** 1.2 M NaCl RIPA buffer

Reagent	Final concentration	Amount
1 M Tris-HCl (pH 8.0)	50 mM	2.5 mL
5 M NaCl	1.2 M	12 mL
10% SDS	0.1%	0.5 mL
10% Triton X-100	1%	5 mL
10% Doc	0.5%	2.5 mL
H_2_O	N/A	27.5 mL
**Total**	**N/A**	**50 mL**

Store at 4°C for up to 3 months

**Table:** Sol A

Reagent	Final concentration	Amount
IACN (≥ 99.97%)	2%	200 μL
H_2_O	N/A	9.79 mL
**Total**	**N/A**	**10 mL**

Store at 4°C for up to 1 month

**Table:** Sol B60

Reagent	Final concentration	Amount
TFA (≥ 99.7%)	0.1%	1Il
ACN (≥ 99.97%)	60%	6 mL
H_2_O	N/A	3.99 mL
**Total**	**N/A**	**10 mL**

Store at 4°C for up to 1 month

**Table:** Sol B30

Reagent	Final concentration	Amount
TFA (≥ 99.7%)	0.1%	10 μL
ACN (≥ 99.97%)	30%	3 mL
H_2_O	N/A	6.99 mL
**Total**	**N/A**	**10 mL**

Store at 4°C for up to 1 month

**Table:** DNA Elution & Reverse crosslinking buffer

Reagent	Final concentration	Amount
5 M NaCl	0.2 M	0.4 mL
10% SDS	1%	1 mL
1X TE	N/A	8.6 mL
**Total**	**N/A**	**10 mL**

Store at room temperature for up to 1 month

**Table:** RNA Elution & Reverse crosslinking buffer

Reagent	Final concentration	Amount
5 M NaCl	0.2 M	0.4 mL
10% SDS	1%	1 mL
1X TE	N/A	8.6 mL
20 mg/mL Proteinase K	0.2 mg/mL	0.1 mL (add just before use)
**Total**	**N/A**	**10 mL**

Buffer without Proteinase K can be stored at room temperature for up to 1 month

***Note:*** Add final concentration 0.2 mg/mL of Proteinase K just before use.

## Step-by-step method details

For the antibody-based *in situ* biotinylation, cultured cells are fixed by paraformaldehyde, permeabilized by detergent, and then incubated with 1^st^ antibodies and HRP-conjugated 2^nd^ antibodies. The *in situ* biotinylation is performed by incubating the cells with biotin phenol and H_2_O_2_. The resulting biotinylated components are purified by avidin pulldown, and purified components are analyzed by MS or NGS. In this protocol, we strongly recommend optimizing the biotinylation condition before sample preparation for MS or NGS. Confirm biotinylation by fluorescently labeled streptavidin before starting full scale sample preparation.

### Optimization of the *in situ* biotinylation condition

**Timing: 2 days**

**Figure 2:**
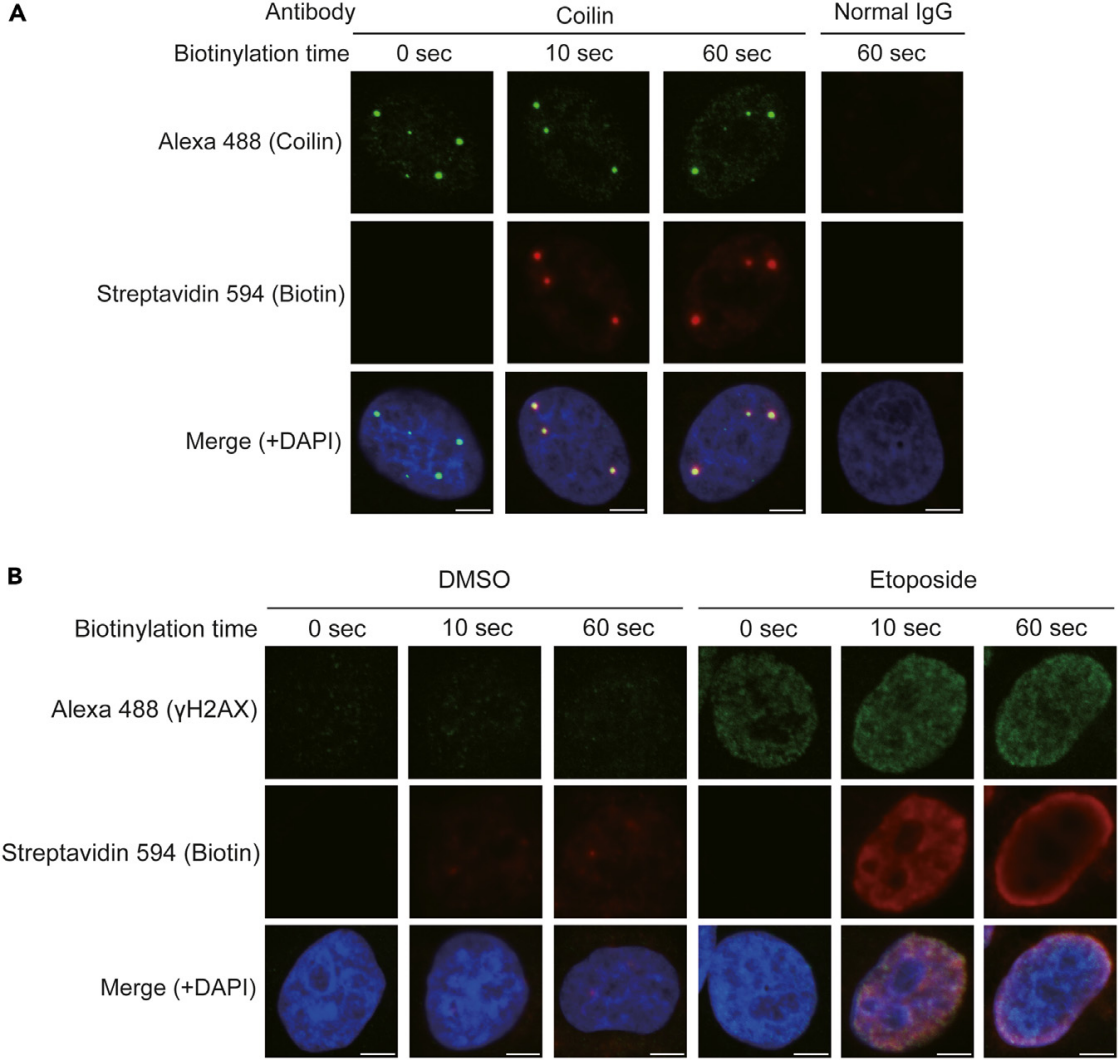
Antibody-based *in situ* biotinylation against Cajal body and γH2AX (A) Titration of the biotinylation condition by streptavidin fluor imaging. HeLa cells were subjected to *in situ* biotinylation using an anti-Coilin antibody. Biotinylation was performed with 500 μM biotin phenol and 0.0015% H_2_O_2_ for 0, 10, and 60 s of reaction time, and visualized with streptavidin-Alexa 594. Endogenous Coilin was visualized with Alexa 488. Scale bar, 5 μm. In these conditions, Cajal bodies (Coilin proximal) were clearly biotinylated with 10 s of biotinylation reaction. For Coilin, a biotinylation reaction of 10–60 s is recommended. (B) γH2AX foci were biotin-labeled using an anti-γH2AX antibody. HeLa cells treated with DMSO or 20 μM etoposide for 1 h were subjected to *in situ* biotinylation using an anti-γH2AX antibody. Biotinylation was performed under the same conditions as described above. Endogenous γH2AX was visualized with Alexa 488. Scale bar, 5 μm. In these conditions, area proximal to γH2AX was clearly biotinylated with 10 s of biotinylation reaction. Reaction time over 60 s was too long, causing biotin signal to not spatially coincide with endogenous γH2AX signal. For γH2AX, a biotinylation reaction of around 10 s or a little longer (e.g., 20 s) is recommended.

Obtaining a good biotinylation signal is necessary for the best results. In this step, you should optimize the biotinylation condition by detecting biotin using fluorescently labeled streptavidin. To assess this, it is necessary to detect your protein of interest using Alexa-labeled 2^nd^ antibody against 1^st^ antibody together with biotin using labeled streptavidin. Confirm that the biotin signal closely overlaps with the immunofluorescence signal of the target protein (Figure 2).

***Note:*** The timing for step 1 is 1 day.

1. Cell culture for fluorescence imaging.
  a. Collagen coating of cover glasses. Before cell seeding, immerse cover glasses in 12-well plates with collagen solution. The collagen solution (Cellmatrix Type I-C, Nitta Gelatin) should be diluted 10-fold with sterile pH 3.0 water, as described in the manufacturer’s instructions.
  b. Remove collagen solution, and air-dry the cover glasses.
  c. Wash the cover glasses with PBS, and add fresh DMEM to wells.
  d. Count cells, and seed the appropriate number of cells on the cover glasses.
  e. Incubate the cells in a CO_2_ incubator at 37°C overnight.

***Note:*** 80% confluency at the time of fixation is suggested for each initial experiment.

**CRITICAL:** Some cell lines, such as HEK293T, easily detach from the cover glass. Handle very carefully to not detach the cells from the cover glass. Alternatively, adjust the coating method for your cell line.

***Note:*** The timing for step 2 is 4 h.

2. Cell fixation, permeabilization, and antibody reaction.
  a. Cell fixation.
    i. Add 0.5 mL of 4% PFA in PBS to each well.
    ii. Incubate for 10 min at room temperature.
    iii. Immediately and carefully remove PFA solution.
    iv. Wash cells with PBS 3 times.
      **CRITICAL:** PFA is harmful. Do not handle without gloves.
  b. Cell permeabilization.
    i. Remove PBS, and add 0.5 mL of 0.5% Triton X-100-containing PBS to each well.
    ii. Incubate for 15 min at room temperature.
    iii. Immediately and carefully wash cells with PBS 2 times.
  c. Blocking to reduce background signal.
    i. Remove PBS, and add 0.5 mL of Blocking buffer to each well.
    ii. Incubate for 1 h at room temperature.
      **Pause Point:** Cells can be stored overnight at 4°C.
  d. 1^st^ antibody reaction. To conserve antibodies, we recommend performing the antibody reactions with droplets on clean parafilm in a humid chamber.
    i. Prepare antibody by diluting with Blocking buffer (e.g., The recommended dilution of anti-Coilin antibody is 1:2,000).
    ii. Prepare clean parafilm in a humid chamber. As a simple method, we lay out a sheet of clean parafilm in a recycled 15 cm dish and place wet kimwipes inside.
    iii. Make 50–100 μL droplets of antibody solution on the clean parafilm.
    iv. Place the cell-attached cover glasses onto the droplets.
    v. Incubate for 1 h at room temperature.
  e. Return the samples to 12-well plates with 0.3% Triton X-100-containing PBS. For cell washing, gently shake the plate for 5 min at room temperature.
  f. Repeat the wash step for a total of 3 times.
    ***Note:*** Be careful to not detach cells from the cover glass during washing.
  g. Completely remove the washing solution, and add 0.5 mL of 2^nd^ antibody solution to 12-well plates.
    ***Note:*** The 2^nd^ antibodies (Alexa fluor-conjugated 2^nd^ antibody and HRP-conjugated 2^nd^ antibody) should be diluted with Blocking buffer. We recommend starting with 1:2000 dilution for Alexa fluor-conjugated 2^nd^ antibody and 1:4,000 dilution for HRP-conjugated 2^nd^ antibody.
    ***Note:*** Alexa fluor-conjugated 2^nd^ antibody and HRP-conjugated 2^nd^ antibody are mixed and incubated at the same time in this protocol.
  h. Incubate for 45 min at room temperature.
  i. Wash cells with 0.3% Triton X-100-containing PBS in 12-well plates.

***Note:*** The timing for step 3 is 30 min.

3. *In situ* biotinylation reaction.
  a. Prepare Biotinylation buffer just before the reaction.
  b. Wash cells with 0.5 mL PBS.
  c. Remove PBS, and add 0.5 mL Biotinylation buffer.
  d. Incubate for 1 min at room temperature.
    ***Note:*** Starting with a 1 min reaction is recommended. Optimization of biotinylation reaction (incubation time, concentration of biotin phenol and H_2_O_2_) is necessary for each experiment.
  e. Immediately wash with PBS 3 times.
    **CRITICAL:** Reaction time directly affects biotinylation intensity. Measure time accurately, and immediately and completely wash out reaction buffer.
    ***Note:*** The timing for step 4 is 1 h.
4. Streptavidin-Alexa fluor labeling and mounting to slide glass.
  a. Prepare streptavidin-Alexa solution just before use. Dilute streptavidin-Alexa 594 1:2,000 with PBS.
  b. Add 0.5 mL of diluted streptavidin-Alexa solution. If DAPI staining is required, add DAPI at a final concentration of 0.5 μM at this step.
  c. Incubate for 30 min at room temperature.
  d. Wash samples with PBS 3 times.
  e. Mount samples to slide glass. Place 10 μL drops of Prolong glass mounting reagent on slide glasses, and carefully put cover glasses on the slide glasses.
  f. Wait until the samples are fixed.

**Pause Point:** Samples can be stored for up to 1 month at 4°C in the dark.

***Note:*** The timing for step 5 is 1–3 h.

5. Detection of biotin signals by confocal microscopy imaging.

***Note:*** Check the biotin signal (intensity and distribution) using microscopy. Use of confocal microscopy is recommended for its high-resolution imaging capacity**.** There are multiple ways to select the best experimental condition. We recommend performing a line plot analysis and choosing the experimental condition with the best signal-noise ratio (Figure 2).

### Preparation of biotinylated cell lysates for MS or DNAs/RNAs for NGS

**Timing: 2–3.5 days**

After optimization of the biotinylation reaction, prepare full-scale lysates for MS or NGS. In many cases, lysate from one 60 mm dish is enough to prepare one sample with replicates for these analyses.

***Note:*** The timing for step 6 is 1 day.

6. Cell culture.
  a. Count cells, and seed an appropriate number of cells in a 60 mm dish.
  b. Incubate the cells in a CO_2_ incubator overnight.

***Note:*** 80%–90% confluency in a 60 mm dish is suggested for each experiment.

***Note:*** The timing for step 7 is 4 h.

7. Cell fixation, permeabilization, and antibody reaction.
  a. Cell fixation.
    i. Add 1.5–2.0 mL of 4% PFA to each dish.
    ii. Incubate for 10 min at room temperature.
    iii. Immediately and carefully remove PFA solution.
    iv. Wash cells with 1.5 mL of PBS 3 times.
      **CRITICAL:** PFA is harmful. Do not handle without gloves.
  b. Cell Permeabilization.
    i. Remove PBS, and add 1.5 mL of 0.5% Triton X-100-containing PBS to each dish.
      **CRITICAL:** Add 10 units/ml of RNase inhibitor for RNA protection.
    ii. Incubate for 15 min at room temperature.
    iii. Immediately and carefully wash cells with PBS 2 times.
  c. Blocking to reduce background signal.
    i. Remove PBS, and add 1.5 mL of Blocking buffer to each dish.
    ii. Incubate for 1 h at room temperature.
      **CRITICAL:** Add 10 units/ml of RNase inhibitor for RNA protection.
      **Pause Point:** Cells can be stored overnight at 4°C.
  d. 1^st^ antibody reaction. Remove Blocking buffer, and add 1.5 mL of diluted antibody solution. Incubate for 1 h at room temperature.
    **CRITICAL:** Add 10 units/ml of RNase inhibitor for RNA protection.
  e. Remove antibody solution, and wash cells with 0.3% Triton X-100-containing PBS. For cell washing, gently shake the dish for 5 min at room temperature.
  f. Repeat the wash step for a total of 3 times.
    ***Note:*** Be careful to not detach cells from the cover glass during washing.
  g. Completely remove the washing solution, and add 1.5 mL of HRP-conjugated 2^nd^ antibody solution to each dish.
    **CRITICAL:** Add 10 units/ml of RNase inhibitor during the 2^nd^ antibody reaction for RNA protection.
  h. Incubate for 45 min at room temperature.
    ***Note:*** The 2^nd^ antibody should be diluted with Blocking buffer.
  i. Wash cells with 1.5 mL of 0.3% Triton X-100-containing PBS 3 times.

***Note:*** The timing for step 8 is 30 min.

8. *In situ* biotinylation reaction.
  a. Prepare Biotinylation buffer just before the reaction.
  b. Wash cells with PBS.
  c. Remove PBS, and add 1.5 mL of Biotinylation buffer.
  d. Incubate for the optimal duration at room temperature.
    ***Note:*** This condition (incubation time, concentration of biotin phenol and H_2_O_2_) should be optimized by image analysis as described above (Figure 2) or by streptavidin-HRP blotting (Figure 3). For streptavidin-HRP blotting, see the steps for cell lysate preparation in the next section, “Preparation of biotinylated lysate.”
    **Figure 3:**
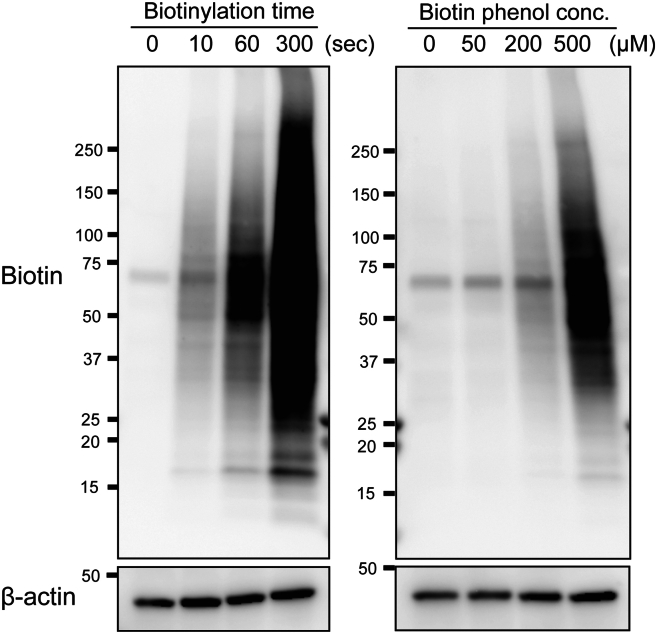
Detection of biotinylated proteins by streptavidin blot HCT116 cells were treated with DMSO or 20 μM etoposide for 1 h and subjected to *in situ* biotinylation using an anti-γH2AX antibody. Biotinylation was performed with 0.0015% H_2_O_2_ and the indicated biotinylation time (0–300 s) or biotin phenol concentration (0–500 μM). Biotinylated proteins were detected by blotting with streptavidin-HRP. The biotin signal was clearly stronger than in the negative controls.
  e. Immediately wash with 1.5 mL of PBS 3 times.
    **CRITICAL:** Reaction time directly affects biotinylation intensity. Measure time accurately, and immediately and completely wash out reaction buffer. Do not handle many samples at the same time.

***Note:*** The timing for step 9 is 1 h.

9. Preparation of biotinylated lysate.
  a. After PBS washing, remove PBS and add 0.5 mL of 1% SDS RIPA Buffer. Use a cell scraper to carefully collect cells in a sterile 1.5 mL tube.
    **CRITICAL:** Add 20 units/ml of RNase inhibitor in 1% SDS RIPA Buffer for RNA detection.
  b. Disrupt cells with a sonicator.
    ***Note:***. We use a Bioruptor homogenizer (Diagenode) with high power, 30 s on and 30 s off for 20 cycles (20 min) at 4°C. Optimize the sonication condition to completely disrupt the cell pellet.
  c. For reverse crosslinking, boil the lysate at 95°C–98°C for 20 min. Make sure that the tube cap does not open while boiling.
    **CRITICAL:** For DNA or RNA detection, skip this reverse crosslinking (boiling) step and immediately go to the next step.
  d. Centrifuge the cell lysate at 15,000 *g* for 10 min at 4°C. Collect the supernatant as biotinylated lysate. The lysate can be stored at −80°C.
    ***Note:*** Detect and optimize the biotinylation signal using streptavidin-HRP (Figure 3). This will take another 5 h.
    **Pause Point:** Cell lysate can be flash frozen with liquid nitrogen and stored at −80°C until performing avidin pulldown.
    ***Note:*** The timing for step 10 is 2.5 h or overnight.
10. Avidin pulldown, beads washing, and elution.
  a. Dilute the biotinylated lysate with the same volume of 2× Dilution buffer (RIPA buffer without SDS), since high concentration of SDS prevents avidin from binding to biotinylated proteins. Now, the total volume of the diluted lysate should be around 1000 μL.
  b. Set aside 50 μL of lysate as an input sample.
  c. Wash avidin beads with 0.5% SDS RIPA buffer two times.
  d. Add 20–30μL of washed avidin beads to the diluted lysate. Rotate the sample at room temperature for 1 h or 4°C overnight.
    ***Note:*** Check the pulldown efficiency, and optimize beads volume and rotation time for each experiment (Figure 4).
    **Figure 4:**
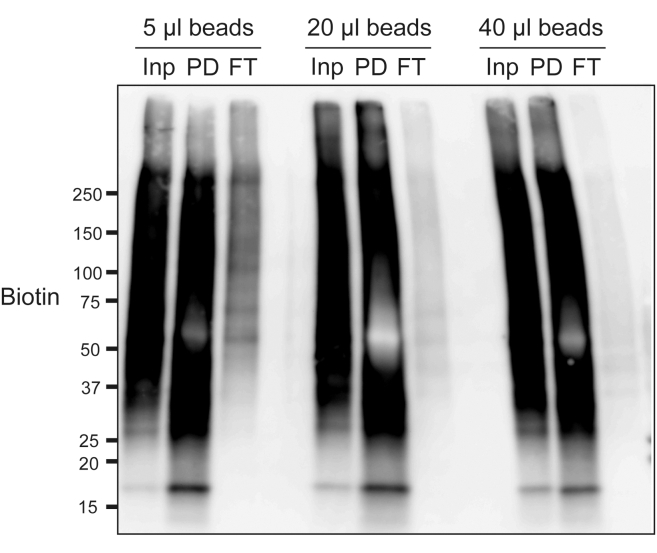
Optimization of avidin pulldown HCT116 cells were treated with 20 μM etoposide for 1 h and subjected to *in situ* biotinylation using an anti-γH2AX antibody. Biotinylation was performed with 0.0015% H_2_O_2_ and 500 μM biotin phenol for 1 min. After lysate preparation, avidin pulldown was performed using the indicated volume of avidin beads for 1.5 h at room temperature. Biotinylated proteins were detected by blotting with streptavidin-HRP. Biotinylated proteins were enriched in the pulldown fraction (PD). Biotin signal was drastically reduced in the flowthrough fraction (FT). In this experiment, 5 μL of beads were not sufficient, and at least 20 μL of beads were needed for a good result.
  e. After incubation, wash beads once each with 0.5% SDS RIPA buffer, 0.5 M NaCl RIPA buffer, 1.2 M NaCl RIPA buffer, and 0.5% SDS RIPA buffer again.
    ***Note:*** For each washing step, centrifuge beads at 2,000 rpm for 1 min, and add 1 mL of washing buffer, followed by 5 min rotation at room temperature.
    ***Note:*** Before the first washing step, a portion of flow-through should be collected to check the pulldown efficiency.
  f. If necessary, confirm enrichment of biotinylated proteins by western blotting.
    i. Separate 1/10 the volume of avidin bead samples as the pulldown sample.
    ii. boil input, flow-through, and pulldown samples for 10 min to denature.
    iii. Detect enrichment of biotinylated proteins using streptavidin-HRP (Figure 4). This will take another 5 h.
      ***Note:*** Use of the proper volume of beads is important. Confirm that most of the biotinylated proteins are captured by avidin beads. Biotin signals in lysate after avidin pulldown (flow-through) should be drastically decreased (Figure 4).
  g. For on-beads trypsin digestion for mass spectrometry analysis, go to “on-beads trypsin digestion and peptide enrichment for mass spectrometry” (steps 11a-h). For qPCR analysis or next generation sequencing for DNA or RNA detection, go to “DNA purification” (steps 12a-h) or “RNA purification” (steps 13a-p).

***Note:*** The timing for step 11 is 1.5 days.

11. On-beads trypsin digestion and peptide enrichment for mass spectrometry.
  a. To completely remove detergent, rinse the beads with distilled water 3 times.
  b. Resuspend the beads with 40 μL of 2 M urea in 50 mM NH_4_HCO_3._
  c. Add 4 μL of 100 mM DTT, and incubate at 37°C for 30 min.
  d. Add 4 μL of 250 mM IAA, and incubate at room temperature for 15 min.
  e. Add 0.5 μL of Trypsin solution (1 μg/μL in 50 mM NH_4_HCO_3_).
  f. Incubate at 37°C overnight. The next day, perform peptide desalting steps.
  g. Peptide desalting steps.
    ***Note:*** For peptide desalting, we usually use self-made stop-and-go-extraction tips (StageTips).^13^ Please see the reference for details on preparation and use of StageTips.^13^ Alternatively, you can use commercial products such as Pierce C18 Spin Tips & Columns (87782 or 87784, ThermoFisher Scientific) or GL-Tips (7820–11200 and/or 7820–11201, GL Sciences).
    i. Put StageTip with centrifuge adapter in a 2 mL tube.
    ii. Tip conditioning step: Add 100 μL of Sol B60 (0.1% TFA, 60% ACN), and centrifuge at 2,000 g for 2 min.
    iii. Equilibration step: Add 100 μL of Sol A (0.1% TFA, 2% ACN), and centrifuge at 2,000 g for 2 min.
    iv. Sample loading: Load sample on StageTip, and pass sample through the tip by centrifugation at 1,000 g.
    v. Washing step: Add 100 μL of Sol A (0.1% TFA, 2% ACN), and centrifuge at 2,000 g for 2 min.
    vi. Repeat the washing step 3 more times.
    vii. Put the tip in a fresh collection tube.
      ***Note:*** A low binding tube such as BM4015 from BMBio ST is recommended for sample collection.
    viii. Elution step 1: Add 50 μL Sol B30 (0.1% TFA, 30% ACN), and centrifuge at 500 g for 5 min.
    ix. Elution step 2: Add 50 μL Sol B60 (0.1% TFA, 60% ACN), and centrifuge at 500 g for 5 min.
    x. Dry out samples using a vacuum concentrator.
  h. Mass spectrometry analysis.
    ***Note:*** Identify proteins by LC-MS/MS. We usually use an Orbitrap Elite, Hybrid Ion Trap-Orbitrap Mass Spectrometer (Thermo Fisher Scientific) coupled with an UltiMate 3000 HPLC system (Thermo Fisher Scientific). Peptide samples were loaded on a trap column (100 μm × 20 mm, C18, 5 μm, 100 Å; Thermo Fisher Scientific) and separated on a Nano HPLC capillary column (75 μm × 18 cm, C18, 3 μm; Nikkyo Technos, Tokyo, Japan) at a flow rate of 300 nL/min. The peptide samples were eluted using a gradient beginning with 2% B (2% ACN with 0.1% TFA) for 0–5 min, then 2%–33% B for 5–120 min, followed by 90% B for 10 min, and finally equilibration with 2% B for 20 min.
    ***Note:*** The timing for step 12 is 1.5 days.
12. DNA purification.
  a. For DNA analysis, wash beads with 1 mL of TE buffer.
  b. Remove TE buffer, and add 200 μL of DNA Elution & Reverse crosslinking buffer.
  c. Incubate at 65°C overnight (12–16 h).
  d. Add RNase A to a final concentration of 0.15 mg/mL. Incubate at 37°C for 30 min.
  e. Add Proteinase K to a final concentration of 0.1 mg/mL. Incubate at 55°C for 2 h.
  f. Purify DNA using the QIAquick PCR Purification Kit MinElute. Elute DNA with 10–20 μL of nuclease-free water.
  g. Determine DNA concentration using Qubit. If necessary, check DNA enrichment at the target locus by qPCR.
  h. Proceed to NGS library preparation for NGS analysis. Perform library preparation in accordance with the manufacturer’s instructions for library prep kits.

**Pause Point:** DNA can be stored at −20°C until performing library preparation.

***Note:*** The timing for step 13 is 3.5 h.

13. RNA purification.
  a. For RNA analysis, wash beads with 1 mL of TE buffer.
  b. Remove TE buffer, and add 200 μL of RNA Elution & Reverse crosslinking buffer.
  c. Incubate at 55°C for 1 h.
  d. Add 500 μL of Trizol LS and mix well by vortexing to stop the reaction.
  e. Incubate the homogenized sample for 5 min at room temperature to allow for the complete dissociation of nucleoprotein complexes, and add 130 μL of chloroform.
  f. Vortex the sample vigorously for 15 s, and incubate it at room temperature for 2–3 min.
  g. Centrifuge the sample at 14,000 *g* for 5 min at 4°C, transfer the aqueous phase to a new tube.
  h. Add 1 μL of GlycoBlue and 2.5× volume of 100% room-temperature ethanol, and vortex for 10 s.
  i. Incubate the sample at room temperature for 10 min.
  j. Centrifuge the sample at 14,000 *g* for 20 min at 4°C.
  k. Remove the supernatant completely, and add 750 μL of 75% (vol/vol) ethanol.
    **CRITICAL:** The RNA pellet is very small. Be careful to not discard the RNA pellet.
    **Pause Point:** The RNA pellet in 75% ethanol can be stored at −80°C for up to 1 week.
  l. Mix the sample by vortexing, and centrifuge it at 14,000 *g* for 5 min at 4°C.
  m. Remove all of the supernatant. Air-dry the RNA pellet for 1 min.
    **CRITICAL:** Do not over-dry the RNA pellet. Over-drying will decrease RNA solubility.
  n. Re-dissolve the RNA pellet in 10–20 μL of nuclease-free water.
  o. Determine RNA concentration using a fluorometer such as Qubit (Thermo Fisher) or Quantus (Promega). If necessary, check RNA enrichment at the target locus by RT-qPCR.
    **Pause Point:** RNA can be flash frozen with liquid nitrogen and stored at −80°C for up to 1 month.
  p. Proceed to NGS library preparation for NGS analysis. Perform library preparation in accordance with the manufacturer’s instructions for library prep kits.

## Expected outcomes

This antibody-based biotinylation technique strongly depends on the performance and specificity of antibodies. The *in situ* biotinylation pattern should be spatially colocalized with the bait protein. Additionally, the biotinylation condition should be optimized by detecting streptavidin-HRP blotting for the best signal-noise ratio. A clear difference between biotinylated lysate and negative control (e.g., biotinylation reaction with normal IgG or no antibodies) should be detected by streptavidin-HRP blotting. The expected yields of protein, DNA, and RNA depend on the type of bait protein and biotinylation condition. Generally, experiments targeting spatially restricted bait proteins, such as Coilin and PML, will generate low yields, while those targeting abundant cellular bait proteins or proteins present throughout cytoplasm and/or nucleoplasm will generate high yields. Note that, as well as protein abundance and localization, the titer of antibody also directly affects the yields. Too weak of a biotinylation signal will result in poor yield. Several biotinylation conditions should be tested to obtain good yields with your bait protein. For DNA and RNA analysis, at least 1 ng and 50 ng, respectively, are required for regular NGS-library preparation.

## Limitations

The first limitation of this antibody-based biotinylation technique is that, due to its high biotinylation efficiency, this method identifies proteins that are present throughout the nucleoplasm such as RNA binding proteins, regardless of their enrichment at nuclear bodies. For this reason, it is not easy to identify nuclear body-specific components. Thus, validation of putative factors as specific nuclear body components will require immunostaining with antibodies against the identified proteins. Second, mass spectrometry analysis identified some false-positive proteins which may be caused by antibody that non-specifically binds to cellular proteins. Careful comparisons of identified proteins in control cells versus cells in which the bait protein is knocked down will help to identify endogenous target proteins. Mass spectrometry signals for true target proteins should be significantly decreased in the knocked-down condition. We found that this comparison helps in identifying true interacting proteins.^1^ Alternatively, to get a list of common contaminants, it would be helpful to perform experiments in parallel targeting other proteins with different cellular localization (e.g., proteins randomly distributed in the nucleoplasm or those that localize to another nuclear body). Finally, biotinylation occurs within an approximately 20 nm radius of HRP rather than at the bait proteins. Considering the size of antibodies (i.e., 1^st^ antibody and HRP-conjugated 2^nd^ antibody), the labeling area may be as far as 60 nm from the bait protein. Because of this, the bait protein itself and its proximal proteins (e.g., direct binding partners) may be undetected, particularly in the case where the bait protein exists as a monomer. Minimizing the distance between the biotinylation radius and the bait protein by fusion of HRP to the 1^st^ antibody is one of the possible solutions for this limitation.

Moreover, the labeling area may depend on the epitope position for 1^st^ antibody. For example, by using a monoclonal antibody that recognizes an N-terminal epitope of the bait protein, the labeling area may be limited to the area proximal to the N-terminus. It may be important to check the epitope position of the antibody. If possible, a mixture of 1^st^ antibodies recognizing different epitopes will resolve the problem of positional bias in the biotinylation area.

## Troubleshooting

### Problem 1

Based on fluorescent streptavidin labeling, biotin signal was not spatially colocalized with the bait protein.

### Potential solution

As shown in Figure 2, too much biotinylation increases non-specific biotin signals. Please optimize the biotinylation condition. In general, a weaker biotinylation condition generates a well colocalized biotinylation pattern and good signal-noise ratio.

### Problem 2

Based on streptavidin-HRP blotting and fluorescent streptavidin labeling, biotinylation signal was too strong or too weak.

### Potential solution

First, please confirm that the antibody works well and targets your protein of interest. Check the antibody performance by immunofluorescence staining. Antibodies with good performance in immunofluorescence staining are strongly recommended for use in this *in situ* biotinylation technique. After confirming the antibody performance in immunofluorescence, next adjust the biotinylation condition, as shown in Figures 2 and 3. Note that a high biotinylation signal does not ensure the success of the experiment. The expected biotinylation signal depends on the target protein (please see “expected outcomes”).

### Problem 3

Expected binding proteins were not detected by western blot or MS.

### Potential solution

This technique sometimes could not detect some well-characterized binding partners. This technique captures various components surrounding the bait protein, regardless of whether there is a direct or indirect interaction. Moreover, since biotinylation occurs in a 20 nm radius of HRP rather than at the bait proteins (see “limitations”), it is possible that the bait protein itself and direct binding partners are not biotinylated. Reducing the distance to the HRP by using HRP-labeled 1^st^ antibody is a potential solution. Alternatively, using a mixture of antibodies recognizing different epitopes may improve the result.

### Problem 4

After avidin pulldown and purification of nucleic acids (DNA or RNA), the yield of nucleic acid is less than the required amount for library preparation.

### Potential solution

The expected yield of samples strongly depends on the performance of the antibody. Additionally, the yield also depends on the function and localization of the target protein. Therefore, the expected yield is highly variable, especially if your target protein is a less abundant one such as some specific transcription factors in the nucleus. In these cases, the yield may be quite low (e.g., 0.5 ng DNA from one 6 cm dish). Although *in situ* biotinylation with a stronger reaction condition generates higher yield, the product may contain more noise. Higher yield does not necessarily lead to better identification. For satisfactory results, please check that the biotin signal is spatially colocalized with and restricted to the target protein. A target-specific biotinylation pattern and good signal-noise ratio is more important than total biotin intensity.

According to the manufacturer’s instructions for the DNA library preparation kit from NEB (NEBNext Ultra II Directional RNA Library Prep Kit for Illumina, E7760), the smallest amount of DNA required for library preparation is 0.5 ng. If the DNA is less than 0.5 ng, in many cases, the cDNA library can be generated by increasing the PCR amplification (up to 15 cycles).

## Resource availability

### Lead contact

Further information and requests for resources should be directed to the lead contact, Hidehisa Takahashi (hide0213@yokohama-cu.ac.jp).

### Technical contact

Technical questions on executing this protocol should be directed to and will be answered by the technical contact, Hidefumi Suzuki (h_suzuki@yokohama-cu.ac.jp).

### Materials availability

Reagents generated in this study are available from the lead contact with material transfer agreements.

### Data and code availability

This protocol did not include new datasets or code.

## Acknowledgments

We thank Miho Uchiumi, Misa Kobayashi, and Kaori Shirouzu for assisting with manuscript preparation. We also thank Hunter Barbee, PhD, from Edanz (https://jp.edanz.com/ac) for editing a draft of this manuscript. This research was supported by AMED-PRIME under grant number 24gm6910006h0002 (H.T.). This work was supported by JSPS KAKENHI grant numbers 16H06279 and 221S0002 (PAGS). This work was supported in part by KAKENHI (20K15718 and 22K15041 to H.S. and 17K19578, 18H02378, 19K22401, 21H05159, 21H02405, and 21K19356 to H.T.) and the Takeda Science Foundation (H.T. and H.S.), Takamatsu Cancer Research Fund (H.T.), Chugai Foundation for Innovative Drug Discovery Science (H.T.), Leukemia Research Fund (H.T.), The Mitsubishi Foundation (H.T.), The Sumitomo Foundation (H.T.), The Ichiro Kanehara Foundation (H.T.), Friends of Leukemia Research Fund (H.T.), Ono Cancer Research Fund (H.T.), Kobayashi Foundation for Cancer Research (H.T.), MSD Life Science Foundation (H.T. and H.S.), The Naito Foundation (H.T.), The Tokyo Biochemical Research Foundation (H.T.), The Uehara Memorial Foundation (H.T.), Yokohama Foundation for Advancement of Medical Science (H.T.), Nakatani Foundation (H.T.), Daiichi Sankyo Foundation of Life Science (H.T.), and SENSHIN Medical Research Foundation (H.S.). We would like to acknowledge BioRender.com for providing the platform for creating the graphical abstract and Figure 1.

## Author contributions

Conceptualization, H.S., R.A., K.N., and H.T.; methodology, H.S., R.A., K.N., and H.T.; formal analysis, R.A.; investigation, H.S., K.L., S.I., and K.A.; resources, H.T.; writing – original draft, H.S. and H.T.; supervision, H.T.; funding acquisition, H.S. and H.T.

## Declaration of interests

The authors declare no competing interests.
